# Pulmonary large cell carcinoma with neuroendocrine morphology shows genetic similarity to large cell neuroendocrine carcinoma

**DOI:** 10.1186/s13000-022-01204-9

**Published:** 2022-02-10

**Authors:** Zuoyu Liang, Weiya Wang, Qianrong Hu, Ping Zhou, Ying Zhang, Yuan Tang, Qian Wu, Yiyun Fu, Xue Li, Yang Shao, Lili Jiang

**Affiliations:** 1 Department of Pathology, West China Hospital, Sichuan University, Chengdu, China; 2 West China School of Medicine, Sichuan University, Chengdu, China; 3 Nanjing Geneseeq Technology Inc, Nanjing, China

**Keywords:** Large cell carcinoma, Large cell neuroendocrine carcinoma, Large cell carcinoma with neuroendocrine morphology, TP53

## Abstract

**Background:**

Large cell neuroendocrine carcinoma (LCNEC) is a high-grade malignant pulmonary neuroendocrine tumour. The distinction of pulmonary large cell carcinoma (LCC) and LCNEC is based on the presence of neuroendocrine morphology and the expression of at least one neuroendocrine marker in at least 10% of tumour cells in the latter. According to the current classification, LCC with neuroendocrine morphology and without neuroendocrine marker expression is classified as LCC. This subgroup we have named LCNEC-null and aimed to analyze its characteristics.

**Methods:**

31 surgical samples resected in West China Hospital of Sichuan University between 2017 to 2021 were collected, including 7 traditional LCCs, 11 LCNEC-nulls and 13 LCNECs. Each case was conducted to immunohistochemistry and 425-panel-NGS.

**Results:**

Compared to other LCCs, detailed analysis of LCNEC-nulls revealed biological features similar to those of LCNECs, especially for immunohistochemistry and molecular analysis: 1. diffusive, coarse granular and high expression of Pan-CK; 2. rare PD-L1 expression; 3. High rate of p53 expression and Rb deficiency 4. abundant genetic alterations are similar to LCNEC. All characteristics above deviated from traditional LCC, indicating they have the same origin as LCNEC. Furthermore, LCNEC could be genetically divided into two subtypes when we reclassified LCNEC-null as LCNEC, and the mutational type and prognosis differed significantly.

**Conclusions:**

We consider that LCNEC-null should be reclassified as LCNEC based on analysis above. In addition, two genetic types of LCNEC with different prognosis also indicate two mechanism of tumour formation.

**Supplementary Information:**

The online version contains supplementary material available at 10.1186/s13000-022-01204-9.

## Background

According to the 2021 WHO definition, large cell carcinoma (LCC) is a non-small cell lung carcinoma (NSCLC) that lacks the cytological, architectural and immunohistochemical (IHC) features of small cell carcinoma, adenocarcinoma, and squamous cell carcinoma [1]. Compared to the 2004 WHO classification, this entity excludes solid adenocarcinoma and nonkeratinizing squamous cell carcinoma. Large cell neuroendocrine carcinoma (LCNEC), basaloid carcinoma and lymphoepithelioma-like carcinoma have been identified as unique entities and removed from LCC. The new classification of LCC transforms a common “waste basket” category into a rare one. Nonetheless, LCC lacks specific alterations and shares some similar mutations with squamous cell carcinoma and adenocarcinoma [2–5]. Rekhtman et al. suggested that null-immunophenotype LCC resembles adenocarcinoma due to its genetic mutation and poor prognosis [6]. Whether LCC is a separate entity or merely a set of poorly differentiated histological types of lung cancer remains in dispute.

As two highly malignant types of pulmonary carcinoma, LCC and LCNEC partially share clinicopathologic features, such as frequent occurrence in the elder, males, and smokers, with a large tumour size, recurrent necrosis, high expression of Ki-67 and unfavorable prognosis. The diagnosis of LCNEC is based on the presence of neuroendocrine morphology and expression of at least one IHC neuroendocrine marker (CD56, synaptophysin (Syn), chromogranin A (CgA)) in at least 10% of tumour cells [1].

LCC and LCNEC are rare histological types, accounting for no more than 1% of all pulmonary carcinomas. Among 5157 surgical resection specimens of pulmonary carcinoma from 2017 to 2021 in the Pathology Department of West China Hospital, Sichuan University, 24 LCCs and 13 LCNECs were diagnosed based on the 2021 WHO classification. Among LCC, we have found 11 with neuroendocrine morphology but without expression of neuroendocrine markers, which we called LCNEC-null. There is scant information about this subtype in the literature. Copin et al. stated LCC with only neuroendocrine morphology or only neuroendocrine immunophenotype should not be classified as LCNEC [7]. The direction of differentiation and growth pattern (cytology and histology) of a tumour largely depends on its precursor cells, whereas the immunophenotype of a tumour can be influenced not only by differentiation degree but also by pathway switching, protein expression, and antibody sensitivity and specificity.

Hence, we hypothesized that LCNEC-null shares common tumourigenesis with LCNEC. In other words, LCNEC-null is a special or poorly differentiated type of LCNEC with negative IHC markers. To address this question, we have performed a multilevel comparison between LCC, LCNEC-null and LCNEC.

## Materials and methods

### Patient material

31 formalin-fixed and paraffin-embedded (FFPE) samples of surgically resected pulmonary large cell carcinoma with or without neuroendocrine differentiation diagnosed from 2017 to 2021 in the Pathology Department of the West China Hospital, Sichuan University were collected, including 7 traditional LCCs (two of them with rhabdoid features), 11 LCNEC-nulls and 13 LCNECs, on the basis of morphology and related IHC staining. These specimens were reviewed by more than two experienced pathologists according to 2021 (5th) Edition of the WHO Classification of Tumours, Thoracic tumours [1]. Relevant data of all cases was collected. After obtaining institutional authorization and the approval from Ethics Committee on Biomedical Research, West China Hospital of Sichuan University (Approval number: 1280), inform consent can be exempted in this retrospective observational research.

### Immunohistochemical staining

4 μm-thick sections extracted from representative FFPE blocks of each case were evaluated for a series of immunohistochemical staining, including cytokeratin 7 (CK7, clone RN7, BIO), TTF-1 (clone 8G7G3/1, ZECA), NapsinA (polyclonal, MXB), P63 (clone UMAB4, BIO), P40 (clone ZR8, MXB), cytokeratin 5/6 (CK5/6, clone D5/16B4, MXB), CD56 (clone UMAB83, BIO), synaptophysin (polyclonal, MXB), chromogranin A (clone EP38, BIO), Pan-CK (clone AE1/AE3, BIO), P53 (clone DO-7, BIO), Rb (clone 13A10, CELNOVTE), Ki-67 (clone MIB-1, DAKO) and PD-L1 (clone 22C3, MERCK). IHC staining was performed by the 2-step Envision procedure using Leica Bond-Max or Roche Ventana autostainer. Related antibodies, together with their clone, dilutions and machine platform was listed on supplementary Table 1. The positive percentage and expression pattern (membrane, cytoplasm or nucleus) were evaluated. Immunophenotype of a tumour was regarded as positive when it expressed moderate or greater intensity in more than 10% of neoplastic cells and reactivity less than 10% cells were considered as negative (excepted for PD-L1). PD-L1 was evaluated by tumour proportion score (TPS), and PD-L1 expression on at least 1% tumour cell was viewed as positive.

### DNA extraction and qualification

According to manufacturer’s instructions, DNA was extracted by a QIAamp DNA FFPE Tissue Kit (Qiagen, Carlsbad, CA, USA) after twice of de-paraffinized by xylene. Extracted DNA was purified and qualified employing the Nanodrop2000 (Thermo), and then using Qubit3.0 (Life Technology) with a dsDNA HS Assay Kit (Life Technology) to quantify DNA.

### Library preparation and sequencing

Amplified and purified DNA Libraries by PCR and then pooled together 1-2 μg of different libraries for targeted enrichment. Hybridization based target enrichment was carried out with NimbleGen SeqCap EZ Hybridization and Wash Kit (Roche). Captured libraries by Dynabeads M-270 (Life Technologies) were amplified in KAPA HiFi HotStart ReadyMix (KAPA Biosystems), followed by purification by Agencourt AMPure XP beads. Customized xGen lockdown probes panel (Integrated DNA Technologies) were used to targeted enrich for 425 predefined genes. The enriched libraries were sequenced on Hiseq 4000 NGS platforms (Illumina) to coverage depths of at least 100x and 300x after removing PCR duplicates for tumour and normal tissue, respectively.

### Bioinformatics analysis

Base calling analysis was used to transfer original image data into raw sequence data, which contained sequence information and corresponding sequencing quality information. Single nucleotide variants (SNVs) and short insertions/deletions (indels) were identified by VarScan2. In-house-developed software was used to detect Copy number variations (CNVs).

### Statistical analysis

All significantly different data between three groups were analyzed using the SPSS 19.0 software (SPSS, Inc., Chicago, IL, USA). Continuous data were evaluated by the independent samples T test, and categorical data by chi-squared test or Fisher’s exact test. Survival analysis was performed by Kaplan-Meier method with the log-rank test using Graphpad Prism 8. *P* < 0.05 was considered as a statistically significant difference.

## Results

### Patient characteristics

With the exception of one LCNEC case, all patients were males with a smoking history (Table 1). The median ages of the LCC, LCNEC-null and LCNEC groups were 61 (range from 41 to 68 years), 64 (range from 48 to 73 years) and 57 (range from 42 to 78 years) years, respectively. LCNEC-nulls and LCNECs presented a slightly smaller tumour size (average size: 4.51 cm and 4.77 cm, respectively) and lower lymph node metastasis (lymph node metastasis: 3 of 11(27.3%) and 5 of 13 (38.5%), respectively) compared to LCCs (average size: 6.74 cm; lymph node metastasis: 4 of 7(57.1%)).

**Table 1:** Contrast of the clinicopathology of LCC, LCNEC-null and LCNEC

	LCC	LCNEC-null	LCNEC
	(*n* = 7)	(*n* = 11)	(*n* = 13)
**Median age**	61 (41–68)	64 (48–73)	57 (42–78)
**Sex**			
Male	7	11	12
Female	**–**	**–**	1
**Smoking history**			
smoker	7	8	12
non-smoker	**–**	3	1
**Tumour size (cm)**	7.5 ± 2.5	5.1 ± 3.2	6.8 ± 4.8
**Lymph node metastasis**	4 (57.1%)	3 (27.3%)	5 (38.5%)
**Stage**			
I	**–**	3	5
II	2	3	1
III / IV	5	5	7
**Pleural invasion**	3 (42.8%)	3 (27.3%)	6 (46.2%)
**Color of cut surface**			
Yellow / Brown	6 (85.7%)	3 (27.3%)	3 (23.0%)
White	1 (14.3%)	8 (72.7%)	7 (53.8%)
Other	**–**	**–**	3 (23.0%)
**Tumour texture**			
Soft	5 (71.4%)	**–**	**–**
Middle	2 (28.6%)	7 (63.6%)	10 (76.9%)
Hard	–	4 (36.4%)	2 (15.4%)

LCC, large cell carcinoma; LCNEC-null, large cell neuroendocrine carcinoma-null immunophenotype; LCNEC, large cell neuroendocrine carcinoma

### CT finding

**Fig. 1:**
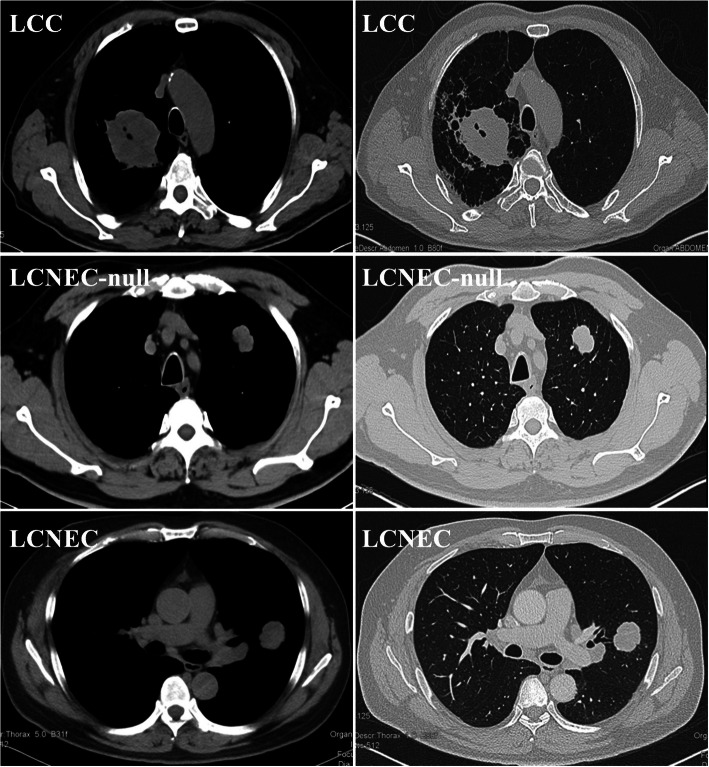
MSCT (Multi-slice computed tomography) of 3 typical cases of each group. A centrally irregular huge mass can be easily found in most LCCs, followed by signs of airway obstruction. By contrast, LCNEC-nulls and LCNECs always show a nodular shadow with lobulated margin in peripheral

We integrated some key CT features of each group, as summarized in Supplementary Table 2. In general, more than half of the cases in our cohort occurred in the bilateral superior lobes, especially the right lung. In the present study, LCCs were more often centrally located (centrally: peripherally = 5: 2), and they (average size: 6.74 cm, range from 5 cm to 10 cm) seemed larger than LCNEC-null (average size: 4.51 cm, range from 1.9 cm to 8.3 cm) and LCNEC tumours (average size: 4.77 cm, range from 2 cm to 11.6 cm). According to evaluation from experienced radiologists, well-defined margins were found in 7 of 11 (63.6%) LCNEC-nulls, 11 of 13 (84.6%) LCNECs and 3 of 7 (42.9%) LCCs. Furthermore, lobulated margins were seen in 10 of 11 (90.9%) LCNEC-nulls, 11 of 13 (84.6%) LCNECs, but only 1 of 7 (14.3%) LCCs. LCCs usually manifested as heterogeneous lesions on CT, corresponding to diffuse necrotic areas in histology (Fig. 1, Supplementary Table 3).

### Macroscopy

The majority of LCNEC-nulls presented a white cut surface (8 / 11, 72.7%) and moderate hardness (7 / 11, 63.6%), which could be seen in more than half of LCNECs (white cut surface: 7 / 13, 53.8%; moderate hardness: 10 / 13, 76.9%). Traditional LCCs were often soft (5 / 7, 71.4%) with yellow or brown surface (6 / 7, 85.7%).

### Morphologic features

Neuroendocrine architecture, including rosettes, organoid structure, nesting with central focal necrosis and peripheral palisading structure, was found in 11 LCNEC-null cases.

Cytologically, the large tumour cells of traditional LCC showed an abundant cytoplasm, a vacuolate nucleus and a prominent nucleolus, lacking a visible intercellular space and presenting a syncytial growth pattern.

Unlike traditional LCC, the smaller hyperchromatic neoplastic cells of LCNEC-null and LCNEC exhibited a slight spindle cell morphology, prominent basophilic cytoplasm, higher nuclear-cytoplasmic ratio and monomorphic round or ovoid nuclei.

**Fig. 2:**
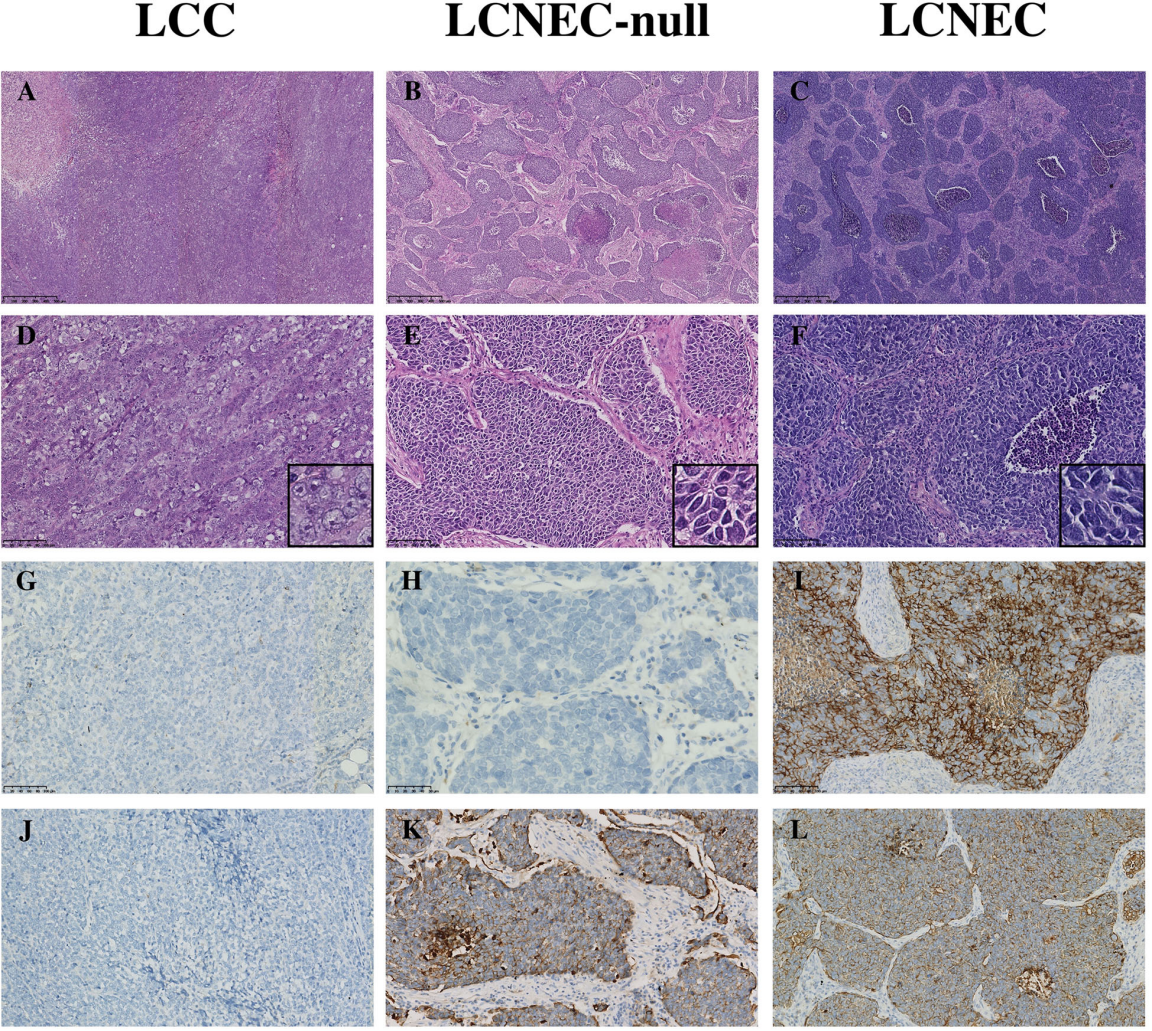
H&E (magnification × 5) (**A, B, C**), H&E (magnification × 20) (**D, E, F**), CD56 (**G, H, I**) and PCK (**J, K, L**) staining sections of typical LCC, LCNEC-null and LCNEC respectively. Unlike traditional LCC, LCNEC-null present neuroendocrine morphology (Nest-like structure with centrally necrosis, surrounded by fibrovascular proliferation, **B, E**) and course-granular of PCK expression (**K**), which is same as LCNEC. The only difference between LCNEC-null and LCNEC is neuroendocrine markers (CD56, **H, L**).

All LCCs contained remarkable diffuse necrosis, but this pattern was only observed in a few bulky LCNECs-null (3 / 11, 27.3%) and LCNEC (4 / 13, 30.8%). In contrast, focal necrosis surrounded by tumour cells and fibrovascular tissue was specifically identified in LCNEC-nulls and LCNECs (Fig. 2 A-F).

### Immunohistochemical profiles

The characteristic immunophenotypes of squamous cell carcinoma (P40, P63 and CK5/6) and adenocarcinoma (TTF-1 and NapsinA) were not observed in LCCs and LCNEC-nulls. No LCC case was positive for neuroendocrine markers (CD56, Syn, CgA). All LCNECs were CD56+, and half of them were positive for Syn (6 of 13, 46.2%), CgA (4 of 13, 30.8%) and TTF-1 (6 of 13, 46.2%). The percentage of Ki-67 expression ranged from 80 to 100% in the hot spot region among the three groups.

Interestingly, remarkable differences in Pan-CK were found among three groups (*p* < 0.001) (Supplementary Table 3). All LCNEC-null and LCNEC tumours showed diffusive, coarse granular expression in the cytoplasm at a high percentage. Only 2 of 7 LCC cases were positive, with rhabdoid cytology and homogeneous positivity in the cytoplasm and membrane (Fig. 2 G-L).

In addition, P53 and Rb were also showed significant distinction: Diffusely strong positivity of P53 could be seen in most LCNEC-nulls (9 / 11, 81.8%) and LCNECs (8 / 13, 61.5%) while all LCCs were negative (*p* = 0.003). No expression of Rb antibody (indicating deficiency) in tumour cells could only be found in 1 of 7 (14.3%) LCCs while 8 of 11 (72.7%) LCNEC-nulls and 8 of 13 (61.5%) LCNECs presented Rb deficiency (*p* = 0.043). Together combined expression of P53 and Rb loss was observed specifically in 6 of 11 (54.5%) LCNEC-nulls and 6 of 13 (46.2%) LCNEC-null.

### Molecular alteration

**Fig. 3:**
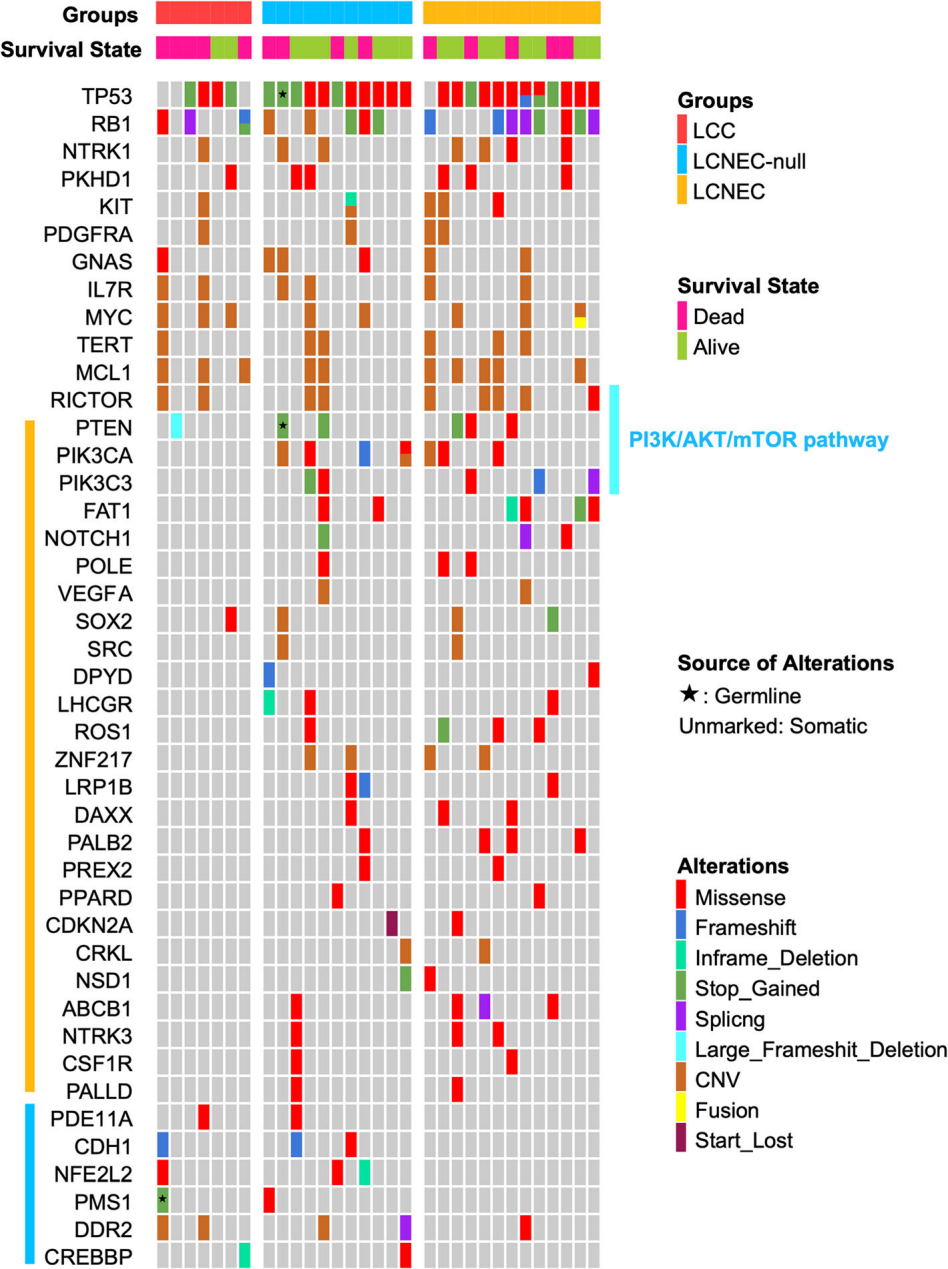
Comparison of clinicopathology and molecular alteration among LCCs, LCNEC-nulls and LCNECs. Molecular alterations we present are based on LCNEC-null. Among 43 genetic alterations detected, the first 12 mutations were shared by three groups, 25 mutations of LCNEC-nulls are similar with LCNECs (marked with orange line), and only 6 are similar with LCCs (marked with blue line)

Mutations detected in more than two cases in all three cohorts are listed in Fig. 3.

Among all 43 oncogenes listed, mutations in the first 12 (TP53, RB1, NTRK1, PKHD1, KIT, PDGFRA, GNAS, IL7R, MYC, TERT, MCL1, RICTOR) were shared by all three groups. Twenty-five genes (marked with orange line) were found in LCNEC-null and LCNEC, including PI3K/AKT/mTOR pathway components. In contrast, mutations in only 6 genes were shared by LCC and LCNEC-null (marked with blue line). Finally, alterations in 21 genes were only detected in LCNEC-null cells (Supplementary Table 4).

All 11 LCNEC-nulls harbored specific genetic alteration which could only be found in LCNEC. While 7 of them harbored LCC-specific mutations. Of note, two unusual oncogene mutation patterns in LCNEC-null should be noted. First, TP53 was inactivated in all eleven cases, with a stop-gain alteration in four of them. Second, concurrent TP53 and RB1 mutation could be found in 5 of 11 LCNEC-nulls.

Additionally, the major receptor tyrosine kinases (RTKs) in the three groups were examined (Supplementary Fig. 1). LCCs and LCNECs presented a different RTK mutation pattern. LCNEC nulls generally have few RTK changes, and most alterations shared by LCNEC.

On average, there were 2.86 CNV alterations per case in LCC, 2.72 in LCNEC-null and 3.38 in LCNEC.

There was no significant difference in overall survival (OS) or progression-free survival (PFS) among LCC, LCNEC-null and LCNEC (Fig. 6 A, B). In contrast to the other two groups, LCC survival was slightly poorer, probably because the patients did not have effective treatment (chemotherapy, radiotherapy and targeted therapy) to control the development of the disease. In addition, we noted that the unusual stop-gain mutation pattern for TP53 in LCNEC-null and LCNEC was closely related to poor prognosis, and this trend was statistically significant (Fig. 6 C).

**Fig. 4:**
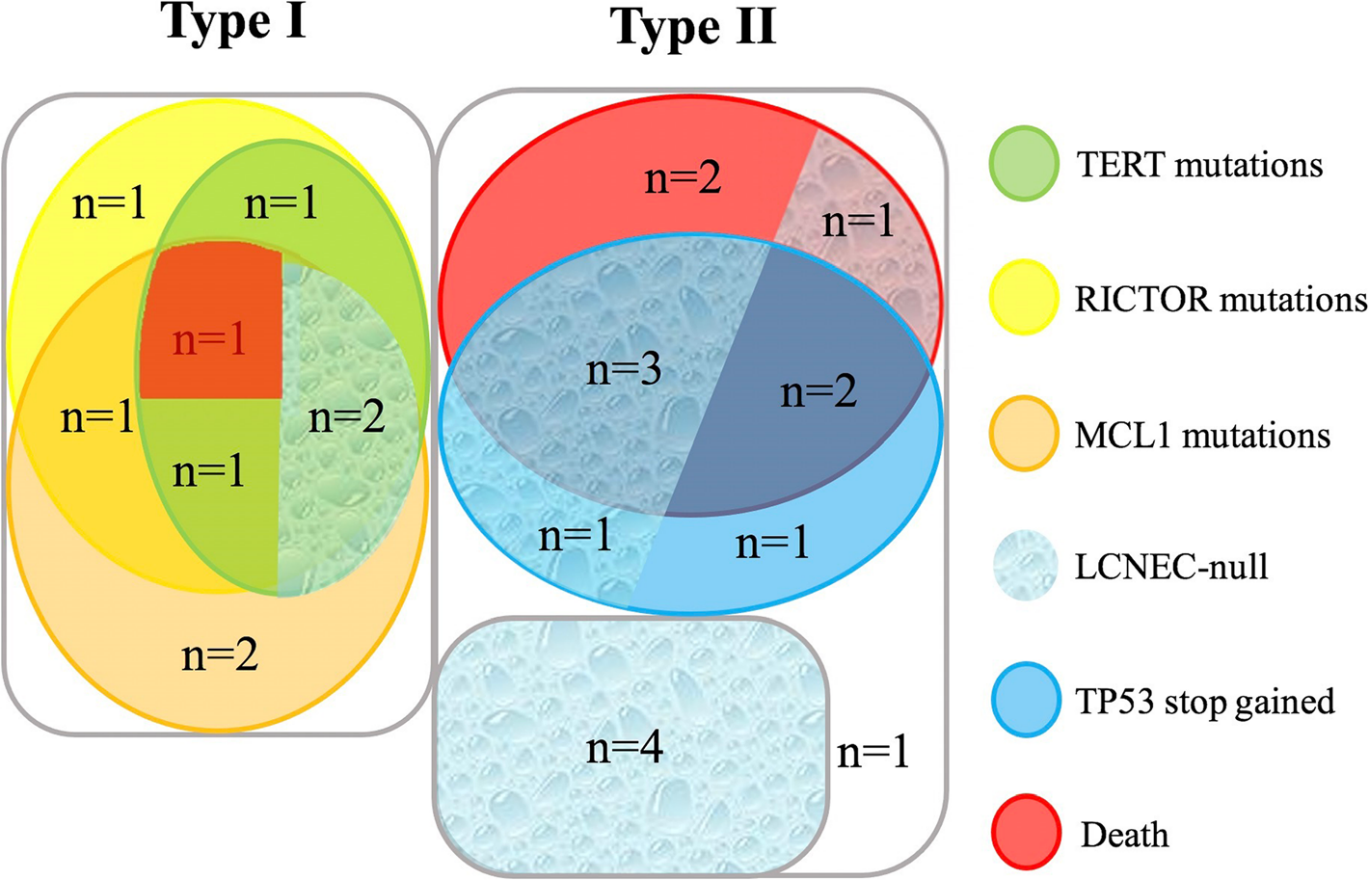
Combined 11 LCNEC-nulls with 13 LCNECs, and then divided them into two different types based on RICTOR and MCL1 mutations. Genetic alterations were marked with circles of different colors, and 9 deceased cases was marked with red circle. Cases of LCNEC-null were filled with droplet background

**Fig. 5:**
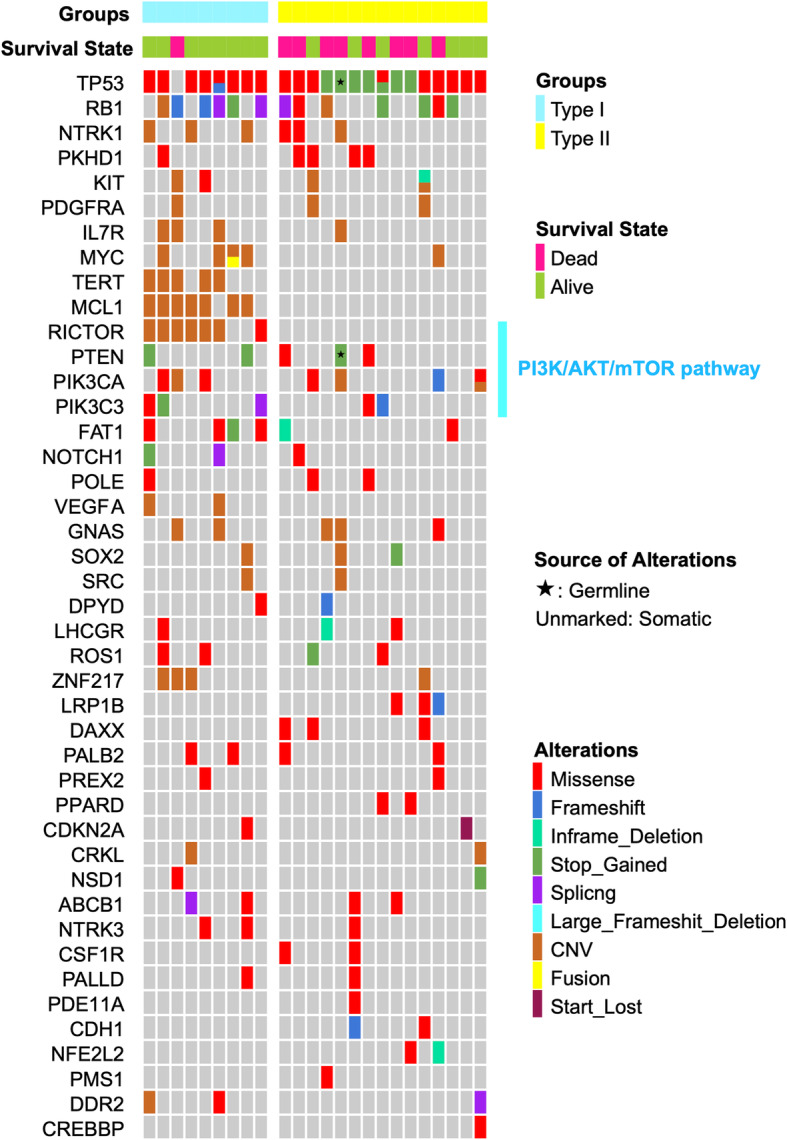
Genetic alterations among two types of LCNEC and LCNEC-null. Type I (*n* = 9) is characterized by high CNV alteration, especially at TERT, RICTOR and MCL1. Type II (*n* = 15) have a high correlation with TP53 stop_gained and poor prognosis

**Fig. 6:**
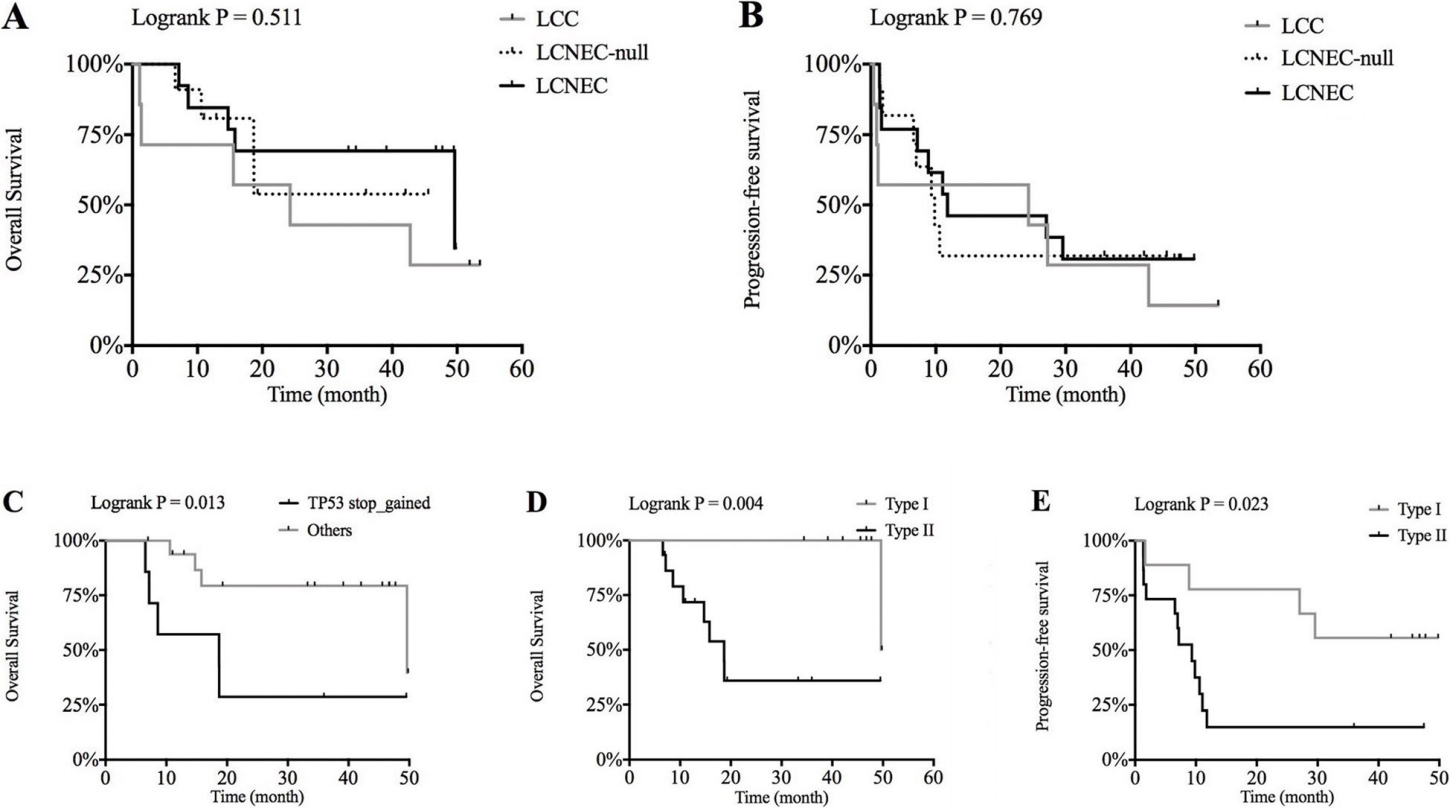
Survival analysis of different groups. **A** Overall survival of LCC, LCNEC-null and LCNEC. **B** Progression-free survival of three groups. **C** Stop_gained mutation pattern of TP53 vs other alterations in LCNEC-null and LCNEC. **D** Overall survival of two types among LCNEC-null and LCNEC. **E** Progression-free survival of two types among LCNEC-null and LCNEC

Interestingly, two significantly different types could be detected when LCNEC-null was combined into the LCNEC group: type I (*n* = 9), characterized by RICTOR or MCL1 mutation; and type II (*n* = 15) with wild-type RICTOR and MCL1. We note type II cases harbored a characteristic high frequency of stop-gain alterations in TP53 (Figs. 4, 5). After a follow-up visit ranging from 7 to 53 months, 8 of 15 patients with type II were deceased because of tumour progression while only one type I patient died (*p* = 0.08). Moreover, type I had more frequent alterations of CNV (averagely 5.6 alterations per case in type I and 1.6 in type II), especially in MCL1 (*n* = 7) and RICTOR (*n* = 6) and TERT (*n* = 5). There were differences in OS and PFS when LCNEC-null was combined with LCNEC, dividing them into type I and type II (Fig. 6 D, E). Although no significant differences of clinical parameters (e.g. age, tumour size, TNM stage) could be found in two types.

## Discussion

Large cell neuroendocrine carcinoma with a null immunophenotype (currently classified as pulmonary large cell carcinoma with neuroendocrine morphology) is extremely rare and therefore adequate studies are missing. In recent years, the majority of studies have concentrated on comparing the characteristics of large cell carcinoma with adenocarcinoma and squamous cell carcinoma [2–6]. A few studies have noted this entity, dating back to the early 2000s, and the special subset called large cell carcinoma with neuroendocrine morphology (in short, LCCNM) by the WHO classification at that time.

Lyoda et al. reported that LCCNM resembles LCNEC in biological behavior by means of univariate and multivariate clinicopathological research [8]. Based on survival analysis, Zacharias et al. found no significant difference in prognosis between LCCNM and LCNEC [9].

However, Peng et al. compared the biological features of LCCNM with LCNEC and pointed out the main difference: the RB pathway is frequently disrupted in LCNEC compared with LCCNM [10]. RB1 mutation could be detected in 8 cases among 13 LCNECs in our cohorts, and 5 of 11 LCNEC-null tumours showed RB1 alteration. Moreover, among 13 LCNEC cases, we found that RB1 inactivation more often occurred in the high CD56 expression group (7 of 8) than in the low CD56 expression group (1 of 5), with a 50% cutoff value (*p* = 0.005). In addition, the high CD56 group more frequently expressed TTF-1 (6 of 8) than the low CD56 group (0 of 5) (*p* = 0.021). There is a potential correlation between CD56, TTF-1 and RB1. However, there were no genetic and prognostic differences when we attempted to separate LCNEC into low and high expression CD56 groups and compare them with LCNEC-null.

In our study all three types (LCC, LCNEC-null and LCNEC) present similar clinical features: the majority were males and smokers, median age about 60, large masses, half of patients in advanced stage. These common characteristics in line with previous studies [11–13].

LCNEC is characterized by peripheral nodules or masses with well-defined and lobulated margins on CT scans, and the mean tumour diameter ranges from 32 to 50 mm, which is highly consistent with our study [14–16]. In our series, the CT appearance was similar between LCNEC-null and LCNEC. Nevertheless, LCC showed completely different CT features, often manifesting as a central enormous mass with a poorly defined border and sometimes showing a spiculate sign.

The dissimilar expression pattern of Pan-CK in the three groups (frequent, diffuse and coarse-granular expression in LCNEC-nulls and LCNECs; rare, focal and fine-granular positive in LCCs) supports that LCNEC-null is different from traditional LCC and more resembles LCNEC.

A low PD-L1 positivity rate in LCNEC-null and LCNEC but a high rate of PD-L1 expression in LCC could be found in our data, in line with Chan’s report [4].

In contrast to LCC, the high Pan-CK and low PD-L1 expression in LCNEC-null and LCNEC probably reflect the relatively good differentiation of these two types.

Based on published data, the two most common alterations in LCC were TP53 and KRAS [3]. Data from these studies, as well as our data for LCNEC-null, are presented in Table 2. As a whole, LCNEC has a higher rate of TP53 mutation and a lower rate of KRAS mutation compared to LCC (TP53, *p* = 0.068; KRAS, *p* = 0.068). All 11 LCNEC-nulls showed TP53 missense or stop_gained, but KRAS mutation could not be detected in them.

**Table 2:** Comparison of the mutation frequency of TP53 and KRAS in different literature

	TP53	KRAS
Rekhtman et al. (LCC) [6]	–	5 / 20 (25%)
Pelosi et al. (LCC) [17]	12 / 20 (60%)	4 / 20 (20%)
Harms et al. (LCC) [3]	11 / 19 (58%)	6 / 19 (32%)
Liu et al. (LCC) [12]	4 / 8 (50%)	2 / 8 (25%)
Zhou et al. (LCC) [18]	3 / 5 (60%)	–
Our data (LCC)	4 / 7 (57.1%)	3 / 7 (42.8%)
**Our data (LCNEC-null)**	**11 / 11 (100%)**	**0 / 11 (0%)**
Our data (LCNEC)	12 / 13 (92.3%)	1 / 13 (7.7%)
Zhou et al. (LCNEC) [18]	12 / 14 (86%)	–
Karlsson et al. (LCNEC) [19]	28 / 32 (87.5%)	2 / 32 (6%)
Naidoo et al. (LCNEC) [11]	–	4 / 17 (24%)
Miyoshi et al. (LCNEC) [20]	55 / 78 (71%)	5 / 78 (6%)
George et al. (LCNEC) [21]	55 / 60 (92%)	6 / 60 (10%)
Rekhtman et al. (LCNEC) [22]	35 / 45 (78%)	10 / 45 (22%)

Lung neuroendocrine tumours are known for their high rate of TP53 and RB1 inactivation [23], especially concurrent TP53 and RB1 mutations. This phenomenon is ubiquitously observed in high-grade lung neuroendocrine tumours [24, 25]. Accordingly, George et al. even classified LCNEC as type I (biallelic TP53 and STK11/KEAP1 alterations) and type II (biallelic mutations of TP53 and RB1, 20]. In our data, comutation of TP53 and RB1 occurred more often in LCNEC-null and LCNEC tumours (5 of 11 and 7 of 13, respectively), while only 1 of 7 LCC tumours showed this alteration.

By NGS, the genomic profile of LCNEC shows high similarity to small-cell lung cancer (SCLC). It has been reported that frequent alteration of the PI3K/AKT/mTOR pathway in SCLC is a promising therapeutic target [24]. Miyoshi and colleagues found 12 of 78 (15%) LCNEC cases with genetic alterations in the PI3K/AKT/mTOR pathway. In addition, the authors suggested that this pathway probably plays an important role in LCNEC tumourigenesis [20]. Rekhtman’s group also reported that 22 of 45 (49%) LCNECs present mutations in the PI3K/AKT/mTOR pathway [22]. Zhou et al. reported that PI3K/AKT/mTOR pathway was most enriched pathway in SCLC, followed by LCNEC, and LCC is the least relevant [18]. Few studies report this pathway in LCC because of its rare incidence. In our series, alteration of this pathway in LCCs was generally rare, and most mutations and/or mutational patterns in this pathway were found by LCNEC-nulls and LCNECs. In addition, PIK3CA, the first upstream gene in this pathway, showed a similar missense mutation in one LCNEC and one LCNEC-null: mutation of guanine to adenine at sites 1624 and 1633, respectively, changing glutamic acid to lysine at positions 542 and 545.

Zhou et al. noted that LCNEC harbors a more frequent CNV mutation pattern than LCC [18]. In our data, 2.72 CNV alterations per case in LCNEC-null but 3.38 in LCNEC and 2.86 in LCC were detected. The differential distribution of CNV alterations in LCNEC was more apparent when LCNEC-null was combined with LCNEC, followed by dividing them into type I (RICTOR/MCL1 alteration, *n* = 9) and type II (characterized by higher TP53 stop_gained and poor prognosis, *n* = 15). There were an average of 5.6 CNV alterations in type I but only 1.6 in type II. The most common CNV sites in type I were MCL1 (*n* = 7), RICTOR (*n* = 6) and TERT (*n* = 5). In general, CNV alterations in MCL1, RICTOR and TERT in pulmonary carcinoma always indicate a poor prognosis [26–28]. While our results lead to the opposite conclusion.

The new grouping method also shows a high correlation between MCL1, RICTOR and TERT. In addition, most LCNEC-nulls landed in the middle of the two circles, which indirectly reflects two different mechanisms of tumourigenesis. If the model we constructed is proven in the future, detecting expression of RICTOR, MCL1, TERT, and TP53 at the protein level would be a promising means to predict the prognosis of LCNEC.

We admitted that there are some limitations in our study. For one hand, this is a retrospective study from a single institution with limited number of cases, which was not convincing enough and might influence IHC and molecular results. For the other hand, potentially more sensitive IHC markers as INSM1 was not available in our department.

## Conclusions

Genetic alteration holds the key to illustrating the occurrence and development of a tumour. Due to the limitation of our samples, exploring genetic difference between LCNEC-null and LCNEC with larger samples in future studies would ultimately unveil the genetic code of neuroendocrine expression. If the features of LCNEC-null in our findings are proven, cases of this type of lung cancer should be redefined as pulmonary neuroendocrine tumours, with greater implementation of targeted therapy (PI3K/mTOR inhibitor).

As a whole, our data shows that LCNEC-null shares more biological features with LCNEC than traditional LCC. Our idea still need to be confirmed by the further, multi-institutional studies. It is unknown whether LCNEC-null is a special variant of LCNEC that lacks enough neuroendocrine differentiation or a type of mild neuroendocrine tumour between LCC and LCNEC. Obviously, however, LCNEC-null should be moved away from traditional LCC due to its biological features.

## Supplementary Information

**Additional file 1 Supplementary Fig. 1.** The major receptor tyrosine kinases (RTK) of three groups

**Additional file 2.**

## Data Availability

All data generated or analyzed during this study are included in this published article and its supplementary information files.

## Abbreviations

WHO: World Health Organization
LCC: large cell carcinoma
NSCLC: non-small cell lung carcinoma
IHC: immunohistochemical
LCNEC: large cell neuroendocrine carcinoma
Syn: Synaptophysin
CgA: chromogranin A
LCNEC-null: large cell neuroendocrine carcinoma-null immunophenotype
FFPE: formalin-fixed and paraffin-embedded
SNVs: Single nucleotide variants
indels: insertions/deletions
CNVs: copy number variations
RTKs: receptor tyrosine kinases
OS: overall survival
PFS: progression-free survival
