# Supplementary figures and images for: Pulmonary large cell carcinoma with neuroendocrine morphology shows genetic similarity to large cell neuroendocrine carcinoma
Source: Diagn Pathol. 2022 Feb 10;17:26. doi: 10.1186/s13000-022-01204-9 (PMC8832809; doi:10.1186/s13000-022-01204-9)

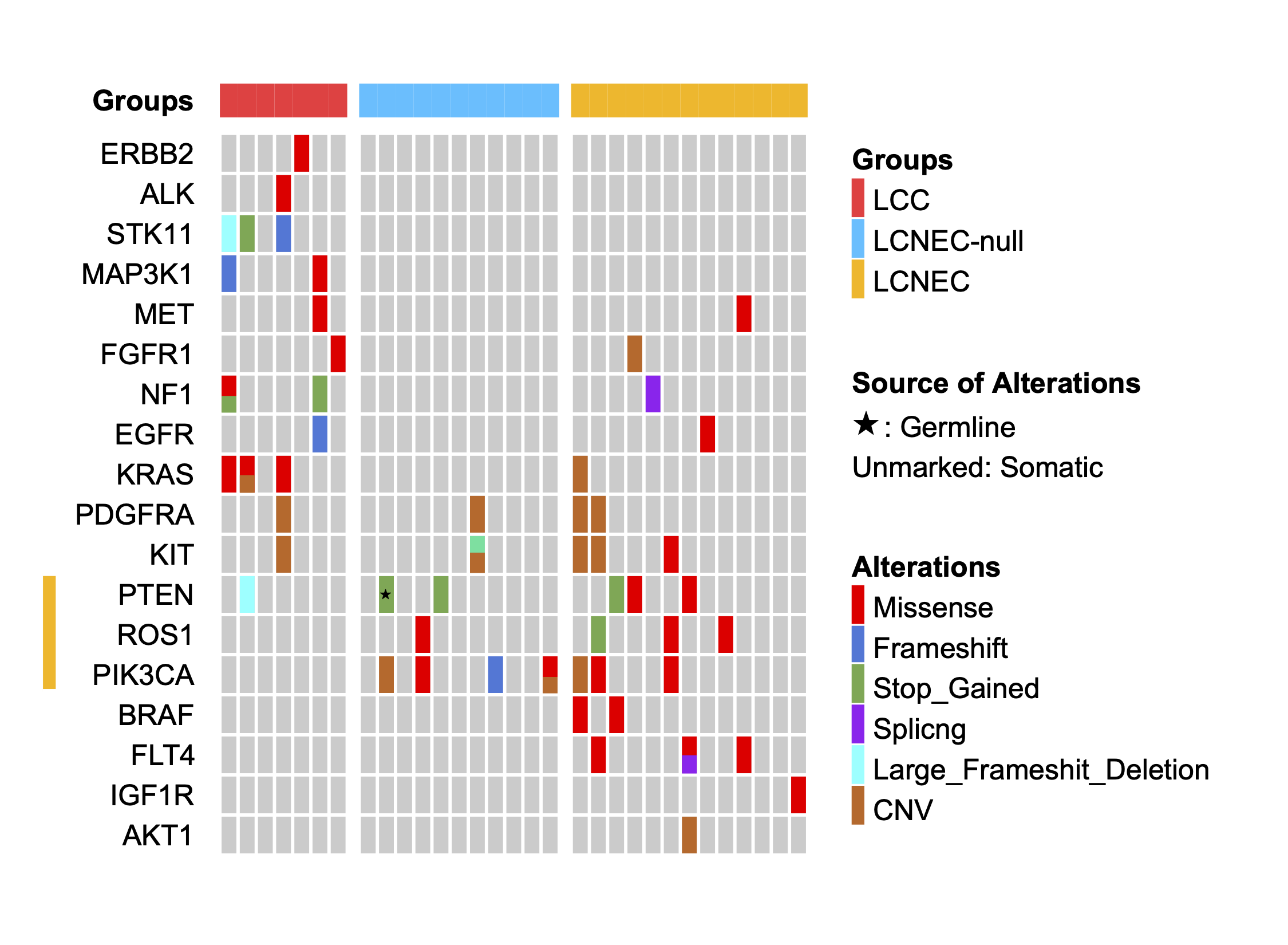

Supplement: Supplementary file 1 — Additional file 1 Supplementary Fig. 1. The major receptor tyrosine kinases (RTK) of three groups [file 13000_2022_1204_MOESM1_ESM.tiff]
