# Supplementary material for: Pulmonary large cell carcinoma with neuroendocrine morphology shows genetic similarity to large cell neuroendocrine carcinoma
Source: Diagn Pathol. 2022 Feb 10;17:26. doi: 10.1186/s13000-022-01204-9 (PMC8832809; doi:10.1186/s13000-022-01204-9)
Supplement: Supplementary file 2 — Additional file 2. [file 13000_2022_1204_MOESM2_ESM.docx]

| **Supplementary Table 1.** Antibodies used for immunohistochemical staining. | | | |
| --- | --- | --- | --- |
| **Antibody** | **Clone** | **Machine platform** | **Dilution** |
| CK7 | RN7 | Roche | 1:100 |
| TTF-1 | 8G7G3/1 | Roche | 1:50 |
| Napsin A | polyclonal | Leica Bond-Max | 1:100 |
| P63 | UMAB4 | Roche | 1:400 |
| P40 | ZR8 | Roche | 1:200 |
| CK5/6 | D5/16B4 | Roche | 1:100 |
| CD56 | UMAB83 | Roche | 1:100 |
| Syn | polyclonal | Roche | 1:200 |
| CgA | EP38 | Roche | 1:200 |
| PCK | AE1/AE3 | Roche | 1:200 |
| P53 | DO-7 | Leica Bond-Max | 1:200 |
| RB | 13A10 | Roche | Ready to use |
| Ki-67 | MIB-1 | Roche | 1:200 |
| PD-L1 | 22C3 | Roche | Ready to use |

| **Supplementary Table 2.** Imaging features of LCC, LCNEC-null and LCNEC. | | | | |
| --- | --- | --- | --- | --- |
|  |  | **LCC** | **LCNEC-null** | **LCNEC** |
|  |  | (n=7) | (n=11) | (n=13) |
| Mean tumour diameter (cm) |  | 6.74 | 4.51 | 4.77 |
| Location | right | 5 (71.4%) | 7 (63.6%) | 9 (69.2%) |
|  | upper | 4 (57.1%) | 6 (54.5%) | 7 (53.8%) |
|  | peripheral | 2 (28.6%) | 10 (90.9%) | 9 (69.2%) |
| Well-defined |  | 3 (42.9%) | 7 (63.6%) | 11 (84.6%) |
| Lobulated margin |  | 1 (14.3%) | 10 (90.9%) | 11 (84.6%) |

| **Supplementary Table 3.** Morphologic features and IHC results in three cohorts. | | | | |
| --- | --- | --- | --- | --- |
|  |  | **LCC** | **LCNEC-null** | **LCNEC** |
|  |  | (n=7) | (n=11) | (n=13) |
| Rosettes |  | 0 (0.0%) | 11 (100.0%) | 13 (100.0%) |
| Organ-like structure | | 0 (0.0%) | 11 (100.0%) | 13 (100.0%) |
| Central focal necrosis | | 0 (0.0%) | 11 (100.0%) | 13 (100.0%) |
| Diffuse necrosis |  | 7 (100.0%) | 3 (27.3%) | 4 (30.8%) |
| PD-L1 | 1% ~ 10% | 1 (14.3%) | 2 (18.2%) | 2 (15.4%) |
|  | > 10% | 5 (71.4%) | 0 (0.0%) | 0 (0.0%) |
| PCK | > 10% | 2 (28.6%) | 11 (100.0%) | 13 (100.0%) |
| P53 | Strong positive | 0 (0.0%) | 9 (81.8%) | 8 (61.5%) |
| Rb | Deficiency | 1 (14.3%) | 8 (72.7%) | 8 (61.5%) |
| CD56 | > 10% | 0 (0.0%) | 0 (0.0%) | 13 (100.0%) |
| Syn | > 10% | 0 (0.0%) | 0 (0.0%) | 6 (46.2%) |
| CgA | > 10% | 0 (0.0%) | 0 (0.0%) | 4 (30.8%) |
| CK7 | > 10% | 1 (14.3%) | 8 (72.7%) | 6 (46.2%) |
| TTF-1 | > 10% | 0 (0.0%) | 1 (9.1%) | 6 (46.2%) |
| NapsinA | > 10% | 0 (0.0%) | 0 (0.0%) | 0 (0.0%) |
| P63 | > 10% | 0 (0.0%) | 0 (0.0%) | 0 (0.0%) |
| P40 | > 10% | 0 (0.0%) | 0 (0.0%) | 0 (0.0%) |
| CK5/6 | > 10% | 0 (0.0%) | 0 (0.0%) | 0 (0.0%) |
| Ki67 | > 50% | 7 (100.0%) | 11 (100.0%) | 13 (100.0%) |

| **Supplementary Table 4.** Detail mutation patterns of 11 LCNEC-nulls. | | | |
| --- | --- | --- | --- |
| Case | Mutation Gene | Mutation type | Mutation site |
| 1 | TP53 | stop_gained | c.497C>G (p.S166X) |
|  | PMS1 | missense | c.106G>T (p.G36C) |
|  | CYP2A13 | inframe_deletion | c.1071_1079delCGAGATCCA (p.H357_I359del) |
|  | LHCGR | inframe_deletion | c.34_51delAAGCTGCTGCTGCTGCTG (p.K12_L17del) |
|  | DPYD | frameshift | c.317delA (p.N106TfsX10) |
|  | RB1 | CNV | . |
|  | GNAS | CNV | . |
| 2 | CTCF | missense | c.1604A>C (p.K535T) |
|  | TERC | CNV | . |
|  | SOX2 | CNV | . |
|  | SRC | CNV | . |
|  | GNAS | CNV | . |
|  | PIK3CA | CNV | . |
|  | IL7R | CNV | . |
|  | NTRK1 | CNV | . |
|  | TP53 (germline mutations) | stop_gained | c.949C>T (p.Q317X) |
|  | PTEN (germline mutations) | stop_gained | c.860C>A (p.S287X) |
| 3 | PKHD1 | missense | c.10980A>T (p.K3660N) |
|  | BRIP1 | missense | c.1489G>C (p.V497L) |
|  | PDE11A | missense | c.2045C>G (p.T682S) |
|  | APC | frameshift | c.1759delA (p.S587AfsX3) |
|  | TP53 | stop_gained | c.1036G>T (p.E346X) |
|  | CSF1R | missense | c.2077C>T (p.P693S) |
|  | CDH1 | frameshift | c.1547_1548delTT (p.F516YfsX20) |
|  | ABCB1 | missense | c.3571G>C (p.V1191L) |
|  | NTRK3 | missense | c.2437C>A (p.Q813K) |
|  | PALLD | missense | c.1473G>T (p.Q491H) |
|  | KMT2B | missense | c.5540C>T (p.P1847L) |
|  | YAP1 | fusion | IGR (downstream MBD2)~YAP1:exon5 |
| 4 | TP53 | missense | c.818G>A (p.R273H) |
|  | PIK3C3 | stop_gained | c.1210A>T (p.K404X) |
|  | TOP2A | missense | c.1897A>G (p.R633G) |
|  | LHCGR | missense | c.569A>G (p.Q190R) |
|  | PKHD1 | missense | c.5411G>C (p.R1804P) |
|  | PIK3CA | missense | c.1633G>A (p.E545K) |
|  | ROS1 | missense | c.1466T>C (p.L489P) |
|  | GATA3 | missense | c.488C>T (p.P163L) |
|  | MLH3 | missense | c.1992A>C (p.K664N) |
|  | GRM8 | stop_gained | c.1857_1858delCGinsTT (p.E620X) |
|  | RB1 | CNV | . |
|  | ZNF217 | CNV | . |
|  | MYC | CNV | . |
|  | TERT | CNV | . |
|  | IL7R | CNV | . |
|  | RICTOR | CNV | . |
|  | MCL1 | CNV | . |
| 5 | TP53 | missense | c.524G>A (p.R175H) |
|  | PTEN | stop_gained | c.919G>T (p.E307X) |
|  | FAT1 | missense | c.6257A>G (p.Y2086C) |
|  | NOTCH1 | stop_gained | c.6169C>T (p.Q2057X) |
|  | POLE | missense | c.2681G>T (p.G894V) |
|  | PTCH1 | stop_gained | c.542C>A (p.S181X) |
|  | PIK3C3 | missense | c.2623G>A (p.V875M) |
|  | PHOX2B | missense | c.547G>C (p.E183Q) |
|  | MCL1 | CNV | . |
|  | BCL2 | CNV | . |
|  | VEGFA | CNV | . |
|  | TERT | CNV | . |
|  | NTRK1 | CNV | . |
|  | RICTOR | CNV | . |
|  | DDR2 | CNV | . |
| 6 | NFE2L2 | missense | c.235G>C(p.E79Q) |
|  | FGFR4 | missense | c.1493G>A(p.R498H) |
|  | PPARD | missense | c.1153C>G(p.L385V) |
|  | APC | stop_gained | c.3184C>T(p.Q1062*) |
|  | TP53 | stop_gained | c.892G>T(p.E298*) |
| 7 | RB1 | stop_gained | c.289G>T(p.E97*) |
|  | TP53 | missense | c.734G>C(p.G245A) |
|  | CDH1 | missense | c.1637C>G(p.A546G) |
|  | LRP1B | missense | c.1096G>C(p.D366H) |
|  | KIT | inframe_deletion | c.1725_1730del(p.Q575_P577delinsH) |
|  | RET | missense | c.45G>T(p.L15F) |
|  | PTCH1 | missense | c.3460G>C(p.A1154P) |
|  | DAXX | missense | c.1318G>A(p.D440N) |
|  | ARID2 | missense | c.412G>A(p.V138M) |
|  | APC | CNV | . |
|  | KIT | CNV | . |
|  | PDGFRA | CNV | . |
|  | ZNF217 | CNV | . |
| 8 | TP53 | missense | c.488A>G(p.Y163C) |
|  | GNAS | missense | c.705C>A(p.D235E) |
|  | RB1 | missense | c.1346G>A(p.G449E) |
|  | LRP1B | frameshift | c.2955del(p.W985Cfs*194) |
|  | NFE2L2 | inframe_deletion | c.94_96del(p.V32del) |
|  | PIK3CA | frameshift | c.2628_2629del(p.L877Tfs*14) |
|  | PALB2 | missense | c.3548A>G(p.Y1183C) |
|  | PREX2 | missense | c.1071G>T(p.L357F) |
|  | MYC | CNV | . |
| 9 | TP53 | missense | c.422G>A(p.C141Y) |
|  | RB1 | stop_gained | c.1759G>T(p.E587*) |
|  | POT1 | missense | c.1094G>T(p.G365V) |
|  | FAT1 | missense | c.7240G>A(p.A2414T) |
| 10 | TP53 | missense | c.536A>G(p.H179R) |
|  | CDKN2A | start_lost | c.-60_75del |
|  | RET | fusion | RET:exon11~ZKSCAN7:exon5 |
|  | NFE2L2 | fusion | KIF13A:exon18~RET:exon11 |
| 11 | TP53 | missense | c.577C>T(p.H193Y) |
|  | PIK3CA | missense | c.1034A>G(p.N345S) |
|  | FOXA1 | missense | c.805G>A(p.E269K) |
|  | BRIP1 | stop_gained | c.2377C>T(p.Q793*) |
|  | CREBBP | missense | c.1686G>A(p.M562I) |
|  | PRKCI | missense | c.452G>T(p.R151L) |
|  | DDR2 | splicing | c.671+2T>C |
|  | NSD1 | stop_gained | c.4045G>T(p.E1349*) |
|  | PTPN13 | intron_variant | c.5112-430_5112-429ins |
|  | CRKL | CNV | . |
|  | PIK3CA | CNV | . |
